# Chemical Composition and Inhibitory Effect of *Lentinula edodes* Ethanolic Extract on Experimentally Induced Atopic Dermatitis in Vitro and in Vivo

**DOI:** 10.3390/molecules21080993

**Published:** 2016-07-29

**Authors:** Eun-Ju Choi, Zee-Yong Park, Eun-Kyung Kim

**Affiliations:** 1 Division of Sport Science, College of Science and Technology, Konkuk University, Chungju 27478, Korea; ooj7990@kku.ac.kr; 2 School of Life Sciences, Immune Synapse Research Center and Cell Dynamics Research Center, Gwangju Institute of Science and Technology, Gwangju 61005, Korea; 3 Division of Food Bioscience, College of Biomedical and Health Sciences, Konkuk University, Chungju 27478, Korea

**Keywords:** atopic dermatitis, *Lentinula edodes*, immunomodulatory, inflammatory cytokines

## Abstract

The ethanolic extract of *Lentinula edodes* was partially analyzed and then characterized for its efficacy in treating atopic dermatitis. Polyphenols were determined to be the major antioxidant component in the extract (6.12 mg/g), followed by flavonoids (1.76 mg/g), β-carotene (28.75 μg/g), and lycopene (5.25 μg/g). An atopic dermatitis (AD) model was established and epidermal and dermal ear thickness, mast cell infiltration, and serum immunoglobulin levels were measured after oral administration of the *L. edodes* extract for 4 weeks. *L. edodes* extract decreased *Dermatophagoides farinae* extract (DFE) and 4-dinitrochlorobenzene (DNCB)-induced expression of several inflammatory cytokines in the ears, cervical lymph nodes, and splenocytes. Consequently, *L. edodes* extract may have therapeutic potential in the treatment of AD attributable to its immunomodulatory effects.

## 1. Introduction

Wild mushrooms are highly valued as a nutritious and palatable food in many societies worldwide [1,2]. They contain high amounts of protein, low total fat levels, and a high proportion of polyunsaturated fatty acids, making them an excellent component of low-calorie diets [3,4]. Furthermore, mushrooms contain dietary fiber, peptides, lectins, phenolics, terpenes, alkaloids, vitamins, and minerals [5,6]. In addition to their nutritional value, many wild edible mushrooms have been investigated for their potential as neutraceutical agents. The antitumor, immunomodulatory, antimicrobial, antifungal, and antiviral properties of wild mushrooms are well established [7,8,9].

*Lentinula edodes* (commonly known as shiitake) is the second most cultivated edible mushroom in the world, representing about 25% of worldwide mushroom production; it has also been used as a food and medicine in South Korea and China for millennia. In the past 10 years, production of *L. edodes* has increased faster than that of any other mushroom species [10]. The bioactive compounds in *L. edodes* have been shown to have anti-tumor and antihypertensive effects [11,12,13,14,15]. *L. edodes* also contain lentinan and vitamin B12, which support the human immune response [16], as well as large amounts of ergosterol and fungisterol. Ultraviolet rays in sunlight can convert bioactive sterol constituents of *L. edodes* into vitamin D, which enhances human resistance to the common cold and other diseases. Meanwhile, there are some reports about inducing effects of *L. edodes* on respiratory symptoms or contact dermatitis [17,18,19,20,21,22,23,24,25]. However, there are also investigations about antiallergenic effects of some mushrooms including *L. edodes* and *Grifola frondosa* [26,27]. Therefore, in this study, we determined the chemical composition of an ethanolic extract of *L. edodes* and its efficacy in the treatment of AD. Chemical analysis included preliminary phytochemical screening of the *L. edodes* ethanolic extract as well as tests to determine carbohydrate and protein quantities, monosaccharide composition, and phenolic, flavonoid, carotenoid, lycopene, and ascorbic acid content. In addition, we used Fourier transform infrared spectroscopy (FT-IR) to further analyze the chemical composition of the *L. edodes* ethanolic extract. To clarify the mechanisms of *L. edodes* ethanolic extract in AD in vitro and in vivo, we investigated its immunomodulatory effects using keratinocyte and murine model, respectively.

## 2. Results and Discussion

### 2.1. Partial Chemical Characterization of Lentinula edodes

Phytochemicals are used in humans to modulate lipid peroxidation involved in atherogenesis, thrombosis and carcinogenesis due to their antioxidant activity and anti-inflammatory action [28,29]. The contents of carbohydrate, monosaccharide, protein, amino acid, and selected phytochemical in the *L. edodes* extract are shown in Table 1.

The polysaccharide and protein concentration of the *L. edodes* extract were 151.43 ± 1.05 μg/g and 205.17 ± 1.44 μg/g, respectively. The monosaccharide constituents of the *L. edodes* extract were primarily glucose (127.78 ± 1.32 μg/mL) and mannose (4.27 ± 0.53 μg/mL). The protein portion of the extract predominantly consisted of glutamic acid (2.8103 g/100 g) and alanine (0.6621 g/100 g).

Polyphenols were the primary constituent of the *L. edodes* extract (6.12 ± 0.04 mg/g), followed by flavonoids (1.76 ± 0.22 mg/g), β-carotene (28.75 ± 0.25 μg/g), and lycopene (5.25 ± 0.04 μg/g). The flavonoids level was higher than that of *L. edodes* from Ethiopia (1.50 mg/g) [30].

**Figure 1:**
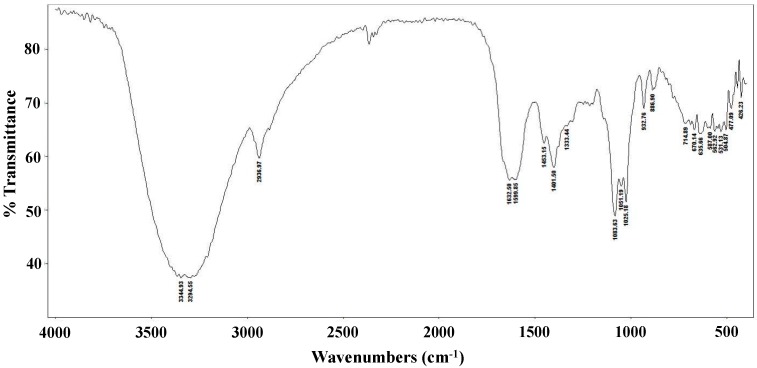
FT-IR spectrum of a crude *Lentinula edodes* ethanolic extract.

The FT-IR spectrum of the *L. edodes* extract is shown in Figure 1. The bands at 3344 and 3294 cm^−1^ indicate hydroxyl stretching of hydrogen bonds and N–H vibrations. The band at 2936 cm^−1^ is attributable to C–H stretching vibrations, whereas the band at 1632 cm^−1^ indicates asymmetric carboxylic acid group vibrations. The peak at 1401 cm^−1^ is associated with the typical stretching frequencies of OH groups from phenolic compounds. Peaks at 1083, 1051, and 1025 cm^−1^ are associated with C–O–C linkages from intact sugars remaining in the extract [31]. 

### 2.2. Lentinula edodes Attenuated Inflammatory Stress in Stimulated Keratinocytes

**Figure 2:**
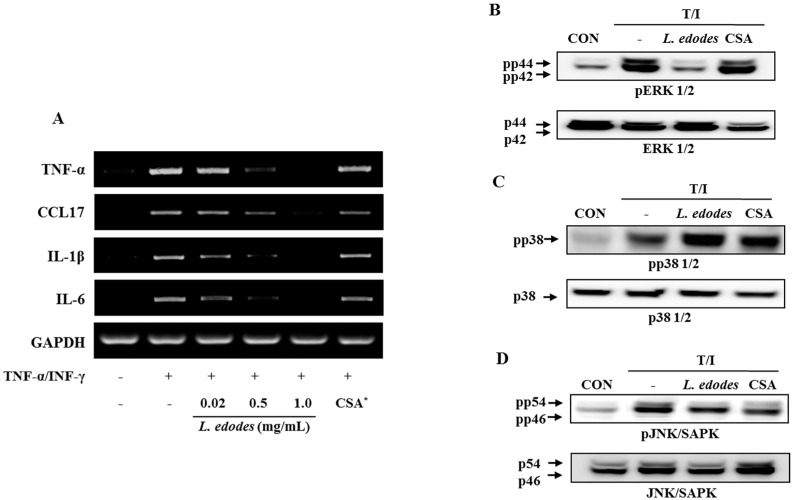
HaCaT cells were pre-incubated with TNF-α (10 ng/mL) and IFN-γ (10 ng/mL) to stimulate the cells. After 6 h of stimulation, cells were harvested and total RNA was isolated. Gene expression levels of pro-inflammatory cytokines (TNFα, CCL17, IL-1β, and IL-6) were measured by conventional PCR (**A**); After 6 h of stimulation, cells were harvested and total protein was isolated. Phosphorylation of ERK1/2 (**B**) and JNK (**D**) was inhibited by treatment with *L. edodes* extract, but that of p38 was not (**C**). The results provided are representative of three independent experiments. * CSA, cyclosporine A (1 μg/mL); +, treatment; −, no treatment.

The effects of *L. edodes* extract on HaCaT keratinocytes co-stimulated by the pro-inflammatory mediators, TNF-α (10 ng/mL) and IFN-γ (10 ng/mL), were assessed using PCR (Figure 2A) and western blot analysis (Figure 2B–D). 

Treatment with *L. edodes* extract effectively reduced TNF-α and IFN-γ-induced mRNA expression of TNF-α, CCL17, IL-1β, and IL-6 in a dose-dependent manner (Figure 2A). These data suggest that treatment with *L. edodes* extract (0.02, 0.5, and 1.0 mg/mL) results in a broad spectrum of inhibitory effects on pro-inflammatory cytokine production in HaCaT keratinocytes stimulated with TNF-α and IFN-γ.

The expression level of mitogen-activated protein kinase (MAP Kinase) in HaCaT cells was also investigated. The keratinocyte was pretreated with *L. edodes* extract at the indicated concentrations for 30 min, after which they were stimulated with TNF-α (10 ng/mL) and IFN-γ (10 ng/mL) for 6 h. Total cell lysates were probed with phospho-specific antibodies for ERK1/2, JNK, and p38. The extent of ERK1/2, JNK, and p38 phosphorylation increased in cells treated with TNF-α and IFN-γ. However, phosphorylation of ERK1/2 and JNK declined after treatment with *L. edodes* extract in relation to the unphosphorylated proteins in TNF-α and IFN-γ co-stimulated HaCaT cells (Figure 2B,D). In contrast, phosphorylation of the p38 protein was unaffected by treatment with *L. edodes* extract (Figure 2C).

### 2.3. Lentinula edodes Supressed Inflammatory Cytokines in a Murine Model

**Figure 3:**
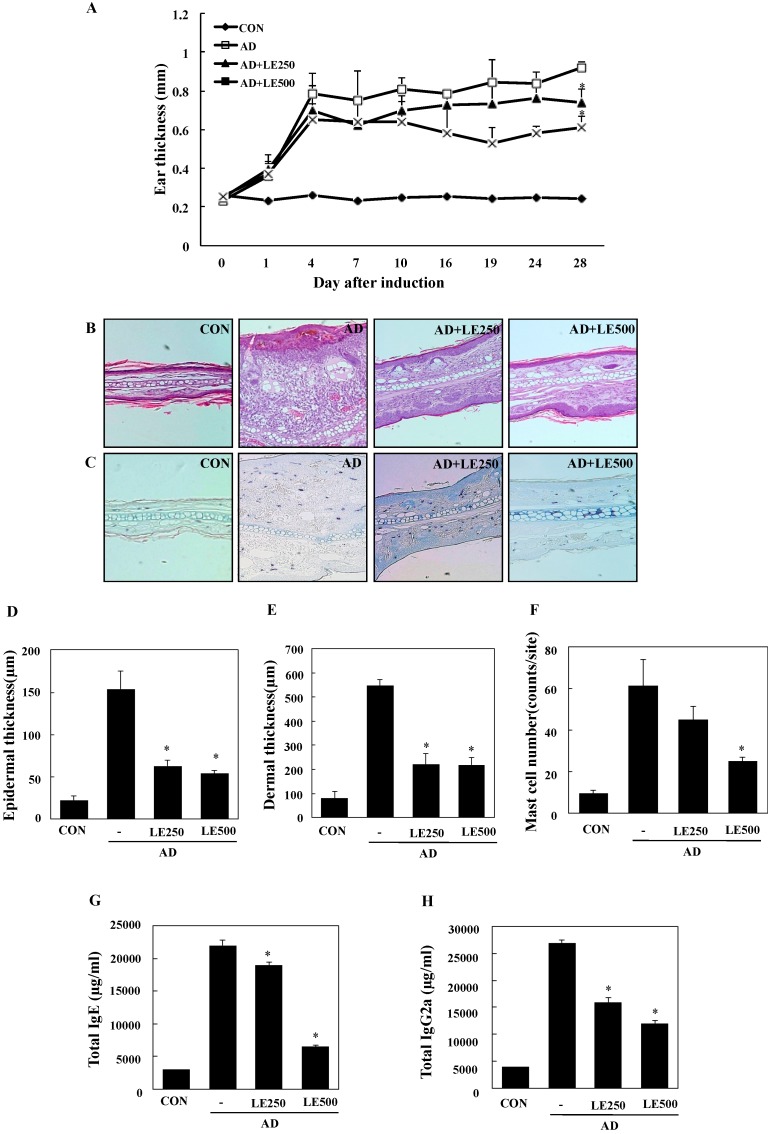
Histopathological and serum analysis to assess the effects of *L. edodes* extract on ear thickness and mast cell infiltration. Ear thickness was measured with a dial thickness gauge every 3 days after 2,4-dinitrochlorobenzene (DNCB) or *Dermatophagoides farinae* extract (DFE) application (**A**); Representative photomicrographs of ear sections stained with hematoxylin and eosin (**B**) or toluidine blue (**C**); epidermal (**D**) and dermal (**E**) thickness was measured using microphotographs of hematoxylin and eosin stained tissue; (**F**) The number of infiltrated mast cells was determined on the basis of toluidine blue staining. Blood samples were collected by orbital puncture at day 28. Plasma IgE (**G**) and IgG2a (**H**) levels were quantified by enzyme-linked immunosorbent assay. Data are presented as the mean ± SD of triplicate determinations. * Significant difference from the value of the DFE/DNCB-treated mice at *p* < 0.05. AD, atopic dermatitis induced by DFE and DNCB treatment. The pictures shown are representative of each group (*n* = 3–6). The original magnification was 100×. CON, control; *L. edodes*, *Lentinula edodes*; AD, atopic dermatitis.

In order to investigate the effects of *L. edodes* extract on AD, a model was established using BALB/c mice by alternately painting DFE and DNCB on both earlobes for 4 weeks [32]. As shown in Figure 3A,B, repeated topical application of DFE/DNCB significantly increased ear thickness. In addition, *L. edodes* extract attenuated the DFE/DNCB-induced increase in ear thickness. DFE/DNCB treatment resulted in remarkable AD lesions, including hemorrhage, edema, excoriation, and scaling that diminished in severity after treatment with *L. edodes* extract (Figure 3B).

Mast cells are important effectors and the primary source of histamine in AD. Therefore, we evaluated the influence of *L. edodes* extract on infiltration of mast cells into the ears. Treatment with *L. edodes* extract reduced the number of infiltrated mast cells in comparison with that induced by DFE/DNCB treatment (Figure 3C,F).

To analyze the effect of *L. edodes* extract on skin hypertrophy and granulocyte infiltration, sections of the ear were stained and observed under an optical microscope. Repeated DFE/DNCB exposure caused pathological changes, including thickening of the epidermis and dermis in the ear tissue of AD mice (Figure 3D,E).

Hyper-production of IgE is associated with a Th2 cellular response and is a primary characteristic of AD. In contrast, IgG2a production is associated with a Th1 response [32]. To determine whether *L. edodes* extract exerts its effects through a Th1 or Th2 response, serum levels of IgE (total and DFE-specific) and IgG2a were measured. Repeated application of DFE/DNCB caused an apparent elevation of total IgE and IgG2a. However, the mice treated with *L. edodes* extract showed significantly reduced serum levels of total IgE and IgG2a than untreated DFE/DNCB-treated mice (Figure 3G,H).

To better understand the mechanisms by which *L. edodes* extract alleviates the AD response, we measured mRNA expression of AD-related inflammatory cytokines in the ear tissue, cervical lymph nodes, and splenocytes by using real-time quantitative PCR. All of the tested cytokines were upregulated in the ear tissue, cervical lymph nodes, and splenocytes of AD mice in comparison to controls.

**Figure 4:**
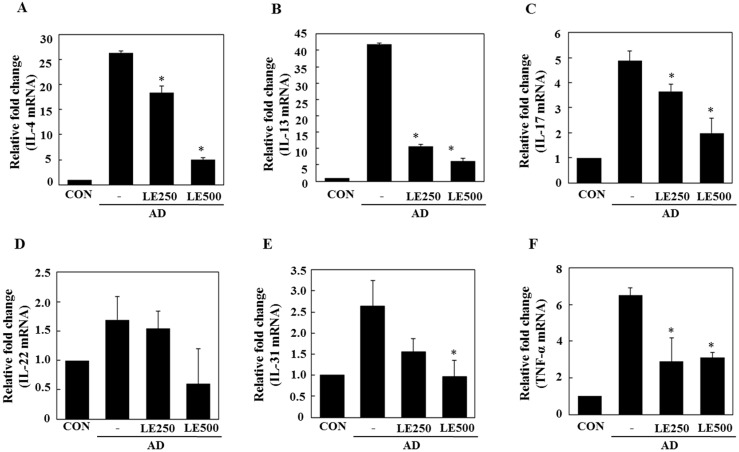
Effect of *L. edodes* extract on the expression of various cytokines in the ear. The ears were excised on day 28 and total RNA was isolated. Quantitative real-time polymerase chain reaction was performed as described in the Materials and Methods. The relative fold change in mRNA for IL-4 (**A**); IL-13 (**B**); IL-17 (**C**); IL-22 (**D**); IL-31 (**E**); and TNFα (**F**) are shown. Data are presented as the mean ± SD of triplicate determinations. * Significantly different from the value of the DFE/DNCB-treated mice at *p* < 0.05. DFE, *Dermatophagoides farinae* extract; DNCB, 2,4-dinitrochlorobenzene; AD, atopic dermatitis induced by DFE and DNCB treatment.

**Figure 5:**
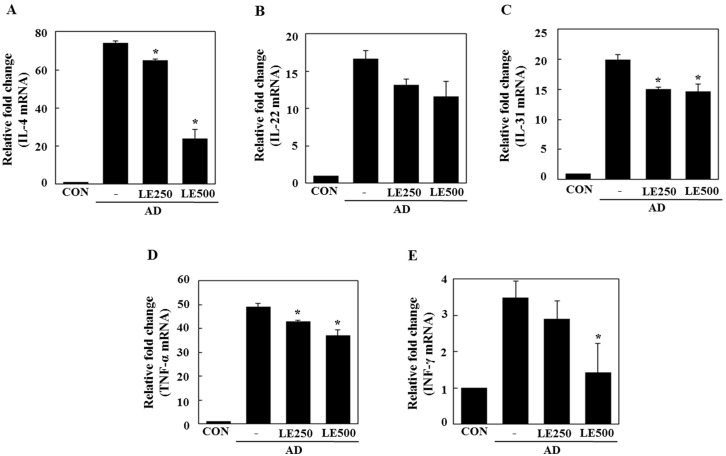
Effect *L. edodes* extract on the expression of various cytokines in the cervical lymph nodes. The cervical lymph node was excised on day 28 and total RNA was isolated. Quantitative real-time polymerase chain reaction was performed as described in the Materials and Methods. The relative fold change in mRNA for IL-4 (**A**); IL-22 (**B**); IL-31 (**C**); TNF-α (**D**); and INF-γ (**E**) are shown. Data are presented as the mean ± SD of triplicate determinations. * Significantly different from the value of the DFE/DNCB-treated mice at *p* < 0.05. DFE, *Dermatophagoides farinae* extract; DNCB, 2,4-dinitrochlorobenzene; AD, atopic dermatitis induced by DFE and DNCB treatment.

**Figure 6:**
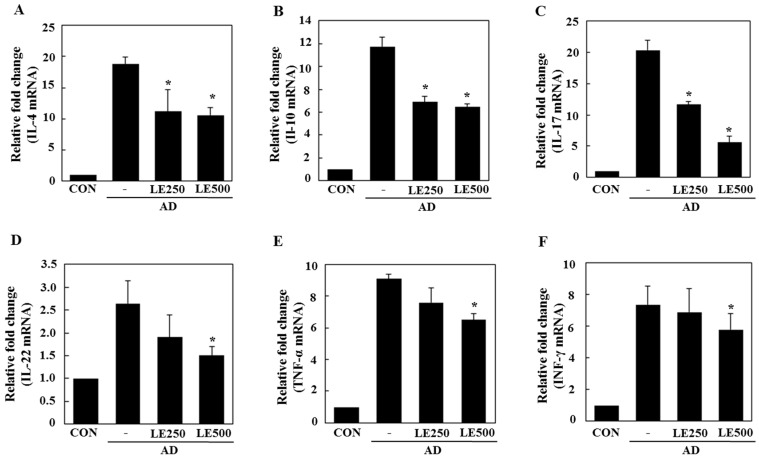
Effect of *L. edodes* extract on the expression of various cytokines in the splenocytes. The splenocytes were separated from spleen on day 28 and total RNA was isolated. Quantitative real-time polymerase chain reaction was performed as described in the Materials and Methods. The relative fold change in mRNA for IL-4 (**A**); IL-10 (**B**); IL-17 (**C**); IL-22 (**D**); TNF-α (**E**); and INF-γ (**F**) are shown. Data are presented as the mean ± SD of triplicate determinations. * Significantly different from the value of the DFE/DNCB-treated mice at *p* < 0.05. DFE, *Dermatophagoides farinae* extract; DNCB, 2,4-dinitrochlorobenzene; AD, atopic dermatitis induced by DFE and DNCB treatment.

In tissue from the ear, *L. edodes* extract significantly inhibited the expression of Th2-related cytokines IL-4, IL-13, IL-17, IL-22, and IL-31, as well as that of TNF-α (Figure 4). In the cervical lymph nodes, *L. edodes* extract reduced the expression of Th2-related cytokines IL-4, IL-22, and IL-31, as well as that of TNF-α and the Th1-related cytokine, IFN-γ (Figure 5). In splenocytes, *L. edodes* extract markedly inhibited the expression of Th2-related cytokines IL-4, IL-10, IL-17, and IL-22, as well as that of TNF-α and the Th1-related cytokine, IFN-γ (Figure 6). It is likely that higher levels of cytokines measured in the ear than that of the cervical lymph nodes and splenocytes are attributable to the direct application of DFE and DNCB to the ear.

Th2 cytokines are highly expressed during the acute stages of AD, whereas Th1 cytokines are predominantly expressed in chronic AD [32]. Our results suggest that *L. edodes* extract inhibits the expression of Th1 and Th2 cytokines in the ears, cervical lymph nodes, and splenocytes, indicating that the therapeutic effects of *L. edodes* extract could be utilized during both the acute and chronic stages of AD.

We assessed the ability of *L. edodes* extract to treat AD-associated pathological lesions and modulate the immune system in BALB/c mice. Alleviation of AD symptoms and stimulation of the immune system by *L. edodes* extract were evaluated by measuring ear thickness, histopathological changes (including mast cell infiltration), serum IgE and IgG2a levels, and gene expression of AD-related inflammatory cytokines in the ear tissue, cervical lymph nodes, and splenocytes from DFE- and DNCB-induced AD mice and controls.

Acute AD lesions are characterized by spongiosis (epidermal intercellular edema) and hyperkeratosis (thickening of the stratum corneum), whereas chronic lesions are characterized by acanthosis (diffuse epidermal hyperplasia) and infiltration of mast cells [33]. In the present study, *L. edodes* extract mitigated the typical histological changes associated with AD, including increases in ear thickness, ulcers, increased epidermal thickness, epidermal hyperplasia, and infiltration of mast cells (Figure 3). Mast cells release several important signaling molecules; histamine, in particular, has potent pro-inflammatory activity [34]. In the present study, oral administration of *L. edodes* extract significantly reduced the severity of the typical histopathological phenomena associated with AD and the number of mast cells infiltrating the skin lesions of AD mice. These data suggest that *L. edodes* extract may directly inactivate mast cells associated with AD.

In AD patients, elevated total IgE and IgG2a levels are commonly detected in response to specific environmental allergens [35]. Historically, a Th1/Th2 imbalance was thought to cause AD symptoms. Th1-mediated inflammation serves to fight infections via IFN-γ, whereas Th2-associated cytokines such as IL-4 and IL-5 are involved in allergic responses and mediate IgE class switching, among other functions [36,37]. In the present study, *L. edodes* extract decreased levels of IL-4 and IL-31, which play an important role in Ig isotype switching. These results imply that *L. edodes* extract suppresses elevated serum IgE levels by decreasing the Th2 response, especially IL-4 production (Figure 4A, Figure 5A and Figure 6A). AD is associated with Th2 expansion in the skin [38]. Recently, Th17 and Th22 were identified as distinct T-cell subsets involved in the pathogenesis of various conditions, including allergic skin diseases [39,40,41,42,43]. A role for IL-17 in allergic skin disease is consistent with the observation that IL-17-deficient mice show impaired contact and delayed-type hypersensitivity responses upon sensitization and challenge with the corresponding allergen [44]. In AD patients, the number of IL-17-positive CD4+ T cells in the peripheral blood correlates well with disease severity [45]. Moreover, IL-17-positive cells infiltrate acute AD lesions [46,47]. In the skin, IL-22 induces keratinocyte proliferation and epidermal hyperplasia. The frequency of IL-22-expressing T cells in the skin of AD patients correlates well with disease severity as well [48,49]. Levels of IL-31 also correlate well with the number of Th2 cells in the skin of subjects with AD [35]. IL-31 transgenic mice developed spontaneous pruritus and hallmarks of AD skin lesions [50]. In our AD model, *L. edodes* extract markedly reduced serum levels of IgE and IgG2a. In addition, the expression levels of Th2-related cytokines IL-4, IL-5, IL-17, IL-22, and IL-31 as well as the pro-inflammatory cytokine, TNF-α, were lower in ear tissue from *L. edodes* extract treated AD mice than that of untreated AD mice (Figure 4). These results indicate that *L. edodes* extract suppresses Th1 and Th2 responses in AD lesions of the ear. In addition, *L. edodes* extract significantly inhibited the expression of TNF-α, Th1-related cytokine IFN-γ, and the Th2-related cytokines IL-4 and IL-22 in the cervical lymph nodes (Figure 5) and splenocytes (Figure 6). Previous studies have reported that some mushrooms suppress pro-inflammatory cytokines, Th1-related cytokines, and Th2-related cytokines in splenocytes [51]. Similarly, we suggest that *L. edodes* extract significantly inhibits the inflammatory response by blocking Th1 and Th2 cell activation in the cervical lymph nodes and splenocytes, as well as in AD lesions of the ear.

## 3. Materials and Methods

### 3.1. Materials

*Cultivated L. edodes* (Sanjo 701) were obtained from the National Institute of Horticultural and Herbal Science of Korea. *L. edodes* specimens were lyophilized and subjected to three 2-h extractions in a 70% ethanol solution. The *L. edodes* extract was filtered (0.25 μm) and lyophilized in a freeze dryer for 5 days. TRIzol reagent for RNA extraction was received from Invitrogen (Carlsbad, CA, USA). Primary antibodies and peroxidase-conjugated secondary antibodies were purchased from Santa Cruz Biotechnology Inc. (Santa Cruz, CA, USA). All other reagents were of the highest grade that was commercially available at the time of the study.

### 3.2. Analysis of Polysaccharides and Monosaccharides 

The total polysaccharide content of the extract was determined by the phenol-sulfuric acid method using d-glucose as a reference [52]. For the polysaccharide analysis, 1 mg of protein-bound polysaccharides was hydrolyzed with 2 M trifluoroacetic acid (TFA) and evaporated. A high-pressure liquid chromatography system (Waters, Milford, MA, USA) with a Sugar-Pak column (Millipore, Tokyo, Japan) and a differential refractive index (RI) detector (RID-6A) was used to detect monosaccharides at 80 °C.

### 3.3. Analysis of Protein Content and Amino Acids 

The protein concentration of the extract was determined using the Bradford method [53]. Bovine serum albumin (BSA, 0.1–1.0 mg·mL^−1^) was used to produce a standard calibration curve. For amino acid analysis, the extract was hydrolyzed in 6N hydrochloric acid in vacuum-sealed tubes at 110 °C for 24 h. The amino acid content of the extract was determined using an amino acid analyzer (L-8900, Hitachi, Tokyo, Japan) with post-column derivatization using ninhydrine. The amino acid analyzer conditions were as follows: ion exchange column (4.6 mm × 60 mm); injection volume, 20 μL; UV detector, VIS1: 570 nm, VIS2: 440 nm. An amino acid standard solution (016-08641, Wako, Osaka, Japan) was used for identification and quantification of amino acids.

### 3.4. Phytochemical Content 

Total phenolic (TP) content of the extract was determined using the recently developed Fast Blue BB (FBBB) method [54]. First, the ethanolic extract was diluted with dimethyl sulfoxide (DMSO) [55,56]. TP analysis then consisted of adding 0.1 mL of 0.1% Fast Blue BB diazonium dye to 1 mL of the diluted sample, followed by the addition of 0.1 mL of 5% NaOH. After 90 min of reaction time, absorbance was measured at 420 nm with a UV/Vis spectrophotometer (UV-1650 PC, Shimadzu, Tokyo, Japan). A standard curve was generated using 15–250 μg·mL^−1^ gallic acid. TP content is expressed as gallic acid equivalents (GAE) per g dry weight (DW) of the extract.

The flavonoid content of the extract was determined by a colorimetric method described by Jia et al. with some modifications introduced by Barros et al. [57,58]. The extract (250 μL) was mixed with 1.25 mL of Milli-Q water (MQ, Temecula, CA, USA) and 75 μL of 5% NaNO_2_ solution. After 5 min, 150 μL of a 10% AlCl_3_ solution was added. After 6 min, 500 μL of 1 M NaOH and 275 μL of MQ were added to the mixture, which was mixed well. The intensity of the solution’s pink color was measured at 510 nm using a UV/Vis spectrophotometer (Shimadzu UV-1650 PC, Kyoto, Japan). (+)-Catechin was used to generate the standard curve (0.022–0.34 mM). Flavonoid content is expressed as mg of (+)-catechin equivalents (CEs) per g of extract.

β-Carotene and lycopene contents were determined according to the method of Nagata and Yamashita [59]. The dried ethanolic extract (100 mg) was vigorously shaken with 10 mL of an acetone-hexane mixture (4:6) for 1 min and filtered through Whatman No. 4 filter paper. Absorbance of the filtrate was measured at 453, 505, and 663 nm. The contents of β-carotene and lycopene were calculated according to the following equations: lycopene (mg per 100 mL) = −0.0458_A663_ + 0.372_A505_ − 0.0806_A453_; β-carotene (mg per 100 mL) = 0.216_A663_ − 0.304_A505_ + 0.452_A453_. The results of these analyses are expressed as μg of carotenoids g^−1^ of the extract.

### 3.5. FT-IR Spectroscopy 

FT-IR spectra were obtained using an Agilent Cary 630 spectrophotometer (Agilent Technologies, Santa Clara, CA, USA) equipped with a Single Bounce Diamond ATR Module for KBr and MicroLab Software plus Resolutions Pro Software (Version 5.0, Agilent Technologies). FT-IR spectra were obtained in the 350–6300 cm^−1^ range at a resolution of 2 cm^−1^ in transmission mode.

### 3.6. Cell Culture 

HaCaT cells were cultured in Dulbecco’s modified Eagle’s medium (DMEM) supplemented with 10% fetal bovine serum and 10 μg/100 mL penicillin/streptomycin in a 5% CO_2_ atmosphere at 37 °C. For analysis by PCR and western blotting, HaCaT cells were pre-incubated with TNF-α (10 ng/mL) and IFN-γ (10 ng/mL) for stimulation. After 6 h of stimulation, cells were harvested and total RNA or protein isolated. 

### 3.7. Animals 

Eight-week-old female BALB/c mice were purchased from Samtako (Osan, Korea) and housed under specific pathogen-free conditions. All experiments were approved by the Institutional Animal Care and Use Committee of Konkuk University (KU14012).

### 3.8. Induction of AD Lesions in the Ear

AD was induced in the mice by repeated local exposure of the ears to *Dermatophagoides farinae* extract (DFE; house dust mite extract) and 2,4-dinitrochlorobenzene (DNCB) as previously described [60]. For the induction of AD, the mice were divided into four groups (control, AD only, AD + *L. edodes* 250 mg/kg, and AD + 500 mg/kg). The surface of both earlobes was stripped five times with surgical tape (Nichiban, Tokyo, Japan). After stripping, 20 μL of 1% DNCB was painted onto each ear; 4 days later, 20 μL of DFE (10 mg/mL) was painted onto each ear. DFE or DNCB treatment was alternated once per week for 4 weeks. Animals were oral administered *L. edodes* (250 or 500 mg/kg) throughout the 4 weeks of DFE and DNCB treatment. 

Ear thickness was measured 24 h after DNCB or DFE application with a dial thickness gauge (ID-C1012XBS, Mitutoyo Co., Kawasaki, Japan). On days 14 and 28, blood samples were collected by orbital puncture. Plasma samples were prepared from the blood samples and stored at −70 °C for further analysis. After the final blood collection, the animals were euthanized and ears, cervical lymph nodes, and splenocyte were removed. Serum immunoglobulin (Ig)E and IgG2a levels were measured on days 14 and 28 after the DFE or DNCB treatment using an IgE enzyme-linked immunoassay kit (Bethyl Laboratories, Inc., Montgomery, TX, USA) according to the manufacturer’s instructions.

### 3.9. Histological Observations 

Excised ears were fixed in 4% paraformaldehyde for 16 h and embedded in paraffin. Thin (6 μm) sections were stained with hematoxylin and eosin (H & E). Epidermal and dermal thicknesses were measured under a microscope. For measurement of mast cell infiltration, skin sections were stained with toluidine blue, after which the number of mast cells was counted in five randomly chosen fields of view.

### 3.10. Analysis of mRNA Expression

For the reverse transcription polymerase (RT)-chain reaction (PCR), the total cellular RNA was isolated from the ear tissue, cervical lymph nodes, and splenocytes of each treatment group using TRIzol according to the manufacturer’s protocol [46]. The first-strand complementary DNA (cDNA) was synthesized using Superscript II reverse transcriptase (Invitrogen). The conditions for RT-PCR were similar ones that have been previously described in related studies [60]. 

For the quantitative PCR, real-time PCR was performed in triplicate using 12.5 μL of SYBR Premix Ex Taq (Takara, Tokyo, Japan) and 2 μL of cDNA as a template in 25 μL of final volume. PCR amplification was preceded by incubation of the mixture for 15 min at 95 °C, and 40 cycles of the amplification step. The denaturation was performed for 30 s at 95 °C; annealing was performed in a transitional temperature range from 58 to 62 °C, with an increase of 0.5 °C per cycle; and the extension was performed for 30 s at 72 °C with fluorescence detection at 72 °C after each cycle. After the final cycle, melting-point analyses of all samples were performed within the range from 65 to 95 °C with continuous fluorescence detection. Target gene mRNA levels were normalized to GAPDH levels using the following formula: relative mRNA expression = 2^−(ΔCt of target gene − ΔCt of GAPDH)^, where *Ct* is the threshold cycle value. In each sample, the expression level of the analyzed gene was normalized to that of GAPDH and presented as a relative mRNA level.

### 3.11. Statistical Analysis

Statistical analysis was carried out using SAS statistical software (SAS Institute, Cary, NC, USA). Multiple group data were analyzed using one-way analysis of variance followed by Dunnett’s multiple range test. All results are expressed as the mean ± standard deviation of comparative fold differences. Data are representative of three independent experiments. The threshold for significance was set at *p* < 0.05.

## 4. Conclusions

In the present study, we demonstrate that *L. edodes* extract ameliorates the development of DFE/DNCB-induced AD symptoms in BALB/c mice by reducing the severity of pathological lesions, production of IgE, and expression of inflammatory cytokines. The evidence provided above indicates that *L. edodes* extract is a potential therapeutic candidate for treating patients with AD. Furthermore, our data suggest that *L. edodes* extract may effectively prevent the occurrence of AD; therefore, it may also be useful as a cosmetic supplement as well as neutraceutical agent.

## Figures and Tables

**Table 1:** Carbohydrates, monosaccharide, total protein, amino acid, and selected phytochemical content in *L. edodes* ethanolic extract.

Components		Content
Total carbohydrates (μg/g)		151.43 ± 1.05
Monosaccharides (μg/mL)	Arabinose	2.57 ± 0.17
	Galactose	1.83 ± 0.19
	Glucose	127.78 ± 1.32
	Mannose	4.27 ± 0.53
	Xylose	2.5 ± 0.51
Total protein (μg/g)		205.17 ± 1.44
Amino acids (g/100 g)	Asp	0.4725
	Thr	0.4435
	Ser	0.3789
	Glu	2.8103
	Pro	0.1789
	Gly	0.3781
	Ala	0.6621
	Val	0.3696
	Ile	0.0963
	Leu	0.1554
	Tyr	0.0711
	Phe	0.1393
	Lys	0.4127
	His	0.1611
	Arg	0.3783
	Cys	0.5109
	Met	0.0284
Phytochemicals	Polyphenols (mg/g)	6.12 ± 0.04
	Flavonoids (mg/g)	1.76 ± 0.22
	β-Carotene (μg/g)	28.75 ± 0.25
	Lycopene (μg/g)	5.25 ± 0.04
